# Effects of variation in coagulation and photochemistry parameters on the particle size distributions in the Venus clouds

**DOI:** 10.1186/s40623-017-0744-x

**Published:** 2017-12-01

**Authors:** Kevin McGouldrick

**Affiliations:** Laboratory for Atmospheric and Space Physics, University of Colorado Boulder, 3665 Discovery Dr., Boulder, CO 80303 USA

**Keywords:** Venus, Atmosphere, Clouds, Meteorology, Dynamics

## Abstract

This paper explores the effects that variation in the coalescence efficiency of the Venus cloud particles can have on the structure of the Venus cloud. It is motivated by the acknowledgment of uncertainties in the measured parameters—and the assumptions made to account for them—that define our present knowledge of the particle characteristics. Specifically, we explore the consequence of allowing the coalescence efficiency of supercooled sulfuric acid in the upper clouds to tend to zero. This produces a cloud that occasionally exhibits an enhancement of small particles at altitude (similar to the upper hazes observed by Pioneer Venus and subsequently shown to be somewhat transient). This simulated cloud occasionally exhibits a rapid growth of particle size near cloud base, exhibiting characteristics similar to those seen in the controversial Mode 3 particles. These results demonstrate that a subset of the variations observed as near-infrared opacity variations in the lower and middle clouds of Venus can be explained by microphysical, in addition to dynamical, variations. Furthermore, the existence of a population of particles exhibiting less efficient coalescence efficiencies would support the likelihood of conditions suitable for charge exchange, hence lightning, in the Venus clouds. We recommend future laboratory studies on the coalescence properties of sulfuric acid under the range of conditions experienced in the Venus clouds. We also recommend future in situ measurements to better characterize the properties of the cloud particles themselves, especially composition and particle habits (shapes).
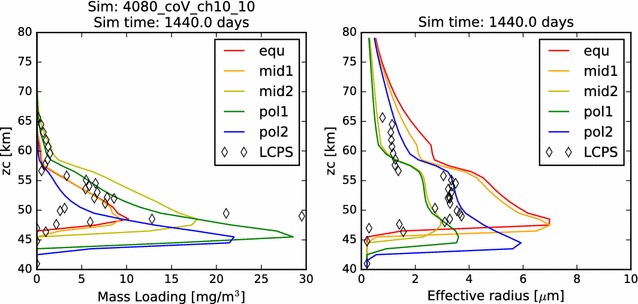

## Introduction

The clouds are a defining characteristic of the atmosphere of Venus. They completely enshroud the planet between the altitudes of about 50 and 70 km. They are composed primarily of sulfuric acid (Sill 1972; Young 1973), but other components have been suggested, including polysulfur (Toon et al. 1982), hydrochloric acid, and phosphoric acid (Krasnopolsky and Parshev 1981). The sulfuric acid is produced via photochemical reactions at altitudes above 60 km (Young 1979). The sulfuric acid vapor nucleates into droplets when supersaturations become large enough, and the particles observed in the upper photochemical clouds tend to be spherical droplets around 1 μm in radius (Hansen and Hovenier 1974), but an additional smaller mode is also regularly seen (Kawabata et al. 1980; Wilquet et al. 2009, 2012). The evaporation of these droplets below an altitude of about 50 km leads to the accumulation of a reservoir of sulfuric acid vapor between about 40 and 50 km, which fuels the condensational clouds of Venus located between about 50 and 57 km, at equatorial latitudes (Toon et al. 1984; James et al. 1997). Below 40 km, the sulfuric acid is thermally decomposed into $\hbox {H}_2$O and $\hbox {SO}_3$ (Krasnopolsky 2007). The clouds are also involved in a radiative-dynamical feedback, whereby cooling from the top of the condensational clouds around 57 km and heating of the base of the clouds by the warmer deep atmosphere at about 50 km lead to the development of convective overturning between about 50 and 57 km that draws vapor from the subcloud region upward where the condensational clouds are located (McGouldrick and Toon 2007).

The most comprehensive description of the Venus cloud particle sizes comes from the Pioneer Venus mission, with subsequent measurements by the VeGa decent probes largely confirming the Pioneer Venus results. In situ observations of the condensational clouds by Pioneer Venus LCPS suggest that two size modes of particles are seen above 57 km, while three modes are seen between 48 and 57 km (Knollenberg and Hunten 1980; Blamont et al. 1986). On the other hand, the Soviet-French Venera-Galley (VeGa) mission to Venus and Comet Halley (Halley in Cyrillic begins with the letters ‘Ga,’ when transliterated back into Latin letters) included two landers and two superpressure balloons. The landers contained particle size spectrometers that were somewhat lower size resolution than the LCPS. They found only a single mode of particles consistent with Mode 1, but with a tail reaching to micron sizes (VeGa-1), and a bimodal distribution lacking Mode 3 particles (VeGa-2). VeGa-2 did, however, detect Mode 3-sized particles near 60 km (Moshkin et al. 1986).

The Pioneer Venus Orbiter Cloud PhotoPolarimeter (OCPP) (Colin and Hunten 1977) confirmed the previous ground-based polarimetric detection (Hansen and Hovenier 1974) of a population of predominantly $1\mu \hbox {m}$, spherical droplets of sulfuric acid as being the primary constituent of the topmost optical depth of the Venus clouds. However, the observations by OCPP also showed that this micron-sized mode of particles generally coexists with a population of submicron particles, which were termed haze (Kawabata et al. 1980). The polar cloud model of Kawabata et al. (1980) suggested a layer of haze overlying a layer of a bimodal population, with the transition occurring at around 40 mb, or about 70 km. However, the Kawabata et al. (1980) equatorial cloud model required an extremely thin ($\tau \approx 0.02$) but nonzero haze layer overlying a predominantly bimodal particle distribution in order to match the polarimetry data.

Kawabata et al. (1980) concluded that the primary constituent of the haze and cloud particles in the topmost optical depth were both sulfuric acid, while noting that the observations, strictly speaking, could not constrain the refractive index in the equatorial region. Subsequent observations indicate a significant level of variability in the smaller mode (Sato et al. 1996; Rossi et al. 2015). However, the sporadic nature of the observations makes it difficult to establish timescales of the variability. One possible mechanism for maintaining a bimodal distribution over long periods of time is to inhibit scavenging of smaller particles via coagulation and coalescence. This could be accomplished, for example, if one or more of the observed modes were solid.

Polarimetric observations are able to characterize only the topmost optical depth of the Venus clouds. Deeper in the atmosphere, other remote sensing techniques, or in situ measurements, must be utilized in order to characterize the particle properties. The Pioneer Venus Large probe Particle Size Spectrometer (LCPS) sampled the clouds of Venus from an altitude of about 65 km down to the surface (though no particles were detected below an altitude of about 31 km).

The LCPS descended at $3^\circ \hbox {S}$ latitude in the Venus atmosphere, so the LCPS data are most correctly compared with the measurements by OCPP at equatorial latitudes. The LCPS data were interpreted to show that two modes of particles were found above an altitude of about 57 km (Knollenberg and Hunten 1980), with modal radii being consistent with those found via polarimetry (Kawabata et al. 1980). Below 57 km, three modes of particles were described by Knollenberg and Hunten (1980). However, the size resolution of the LCPS detector, while excellent 40 years ago, was remarkably crude by today’s standards, leaving the characterization of the true Venus cloud particle size distribution somewhat unconstrained, For example, Toon et al. (1984) suggested that a possible mismatch in the calibration of the detector ranges would have resulted in the so called Mode 3 particles being characterized as having radii slightly larger than the true particles. That is, if the largest detected particles are shifted to slightly smaller radii, then the Mode 3 distribution manifests itself as a large-radius tail of the well-established and well-observed Mode 2 population. The reader is referred to the original papers (Toon et al. 1984; Knollenberg 1984) for further details.

In addition to the Mode 3 controversy, the smallest mode of particles measured by the LCPS is similarly unconstrained. Note from Figure 10 of Knollenberg and Hunten (1980) that the size distribution parameters of the Mode 1 particles defined by Knollenberg and Hunten (1980) are determined from only two measurement bins. That figure shows that a wide range of potential size distributions can be shown to fit the existing data. In order to constrain this degeneracy, Knollenberg and Hunten (1980) postulated that the range of allowable parameters for the Mode 1 particle size distribution would be limited by microphysical processes, namely coagulation timescales. Their Figure 10 shows that distributions with significantly smaller modal radii would require considerably larger number concentration of particles in order to fit the two observational data points. Knollenberg and Hunten (1980) argued that the coagulation timescale would be short enough that such a distribution would be very unlikely. Hence, they settled on the distributions that they published—Mode 1 with a radius of about $0.3\,\upmu \hbox {m}$, and lognormal standard deviation of 2.16—which population is consistent with a coagulation loss timescale of about 60 days. Note that if the coagulation efficiency of these submicron particles is reduced, then one of the arguments against the existence of the controversial ‘Mode 0’ particles (Tomasko et al. 1980) in the upper part of the Venus cloud system also goes away.

However, if the coalescence efficiency was significantly lower than assumed by Knollenberg and Hunten (1980), then these smaller populations of particles could no longer be ruled out. McGouldrick et al. (2011) suggested that frozen, or at least supercooled and glass-like, particles of sulfuric acid were consistent with the structure of the atmosphere of Venus above about 57 km (*i.e.*, in the upper clouds and hazes). If frozen or glass-like particles were to exist at these altitudes, their coalescence properties would differ from those of liquid sulfuric acid, and hence, the expected size distributions of the cloud particles may vary. Furthermore, as suggested by McGouldrick et al. (2011), the presence of a significant population of solid particles in the upper clouds can create conditions that are capable of producing intracloud lightning between the photochemical and condensational clouds of Venus.

Since temperature-dependent coalescence, as suggested by McGouldrick et al. (2011), would most likely be inhibiting coagulation only in the photochemical clouds above about 57 km (the altitude above which temperature first drops below the nominal melting point of sulfuric acid at equatorial latitudes on Venus), we extend the domain of my model to 80 km. This necessitates the inclusion of a simplified set of chemical reactions and the inclusion of a binary homogeneous nucleation scheme.

In this paper, we investigate the effect that changes in the coalescence properties of Venus cloud droplets would have on the appearance of the Venus cloud system. First, we attempt to reproduce the results of my 2007 model (McGouldrick and Toon 2007, 2008a, b), since we have made several improvements and adjustments in the model since those papers were published. We then demonstrate some of the expected latitudinal variability in the clouds, driven solely by variations in the vertical structure of the atmosphere. Next, we perform a series of sensitivity tests, exploring the effects of the parameters chosen for the simplified photochemical production and loss rates. Finally, we present a comparison of simulations in which the only varied parameter is the coalescence efficiency. We show that significant and measurable variations in the overall mass loading, particle effective radius, and particle size distribution can be obtained by these changes.

To reduce the number of variables in the current simulations, we turn off the radiative-dynamical feedback that was demonstrated in McGouldrick and Toon (2007). A follow-up paper is under preparation in which the radiative transfer again interacts with the clouds, in both the radiative-dynamical feedback and in the form of insolation-dependent photochemistry. All of this is part of a long-term effort to simulate the interaction between the microphysics and the atmospheric dynamics of Venus using a three-dimensional model such as a GCM or one capable of simulating mesoscale dynamical processes.

## Model description

The model used leans heavily on heritage from the work of McGouldrick and Toon (2007, 2008a, b). It is based on the Community Aerosol and Radiation Model for Atmospheres (CARMA), which was first described in a NASA Technical report, and then by Toon et al. (1988). The model has been updated to incorporate the changes included in version 2.3, but not the changes included in version 3.0. Most of the changes between version 2.3 and 3.0 are either improvements in efficiency that result from the update to Fortran 90 from Fortran 77, or related to the substepping of the fast microphysics. Since my existing model already made a number of improvements to the substepping, and since the CARMA 3.0 substepping was deemed insufficient for my needs, the decision was made to instead build upon my existing model. The initial conditions defined for this model are summarized in Table 1. A summary of the simulations performed is included in Table 2.

We prepare two sets of models. Both have a lower boundary of 40 km. One set has an upper boundary of 60 km, with 1 km vertical resolution. A second set of models extends this domain to 80 km by introducing an additional ten layers having 2 km vertical resolution. The range of particle sizes simulated here ranges from 1 nm to 800 micron in 60 logarithmically spaced radius bins in which the mass ratio of each larger bin to the next smaller is 2:1.

Finally, we note that this is a one-dimensional column model. This has the utility of being most easily incorporated in the future into more sophisticated atmospheric dynamics models, but means that a number of important dynamical drivers of the clouds are necessarily missing from consideration. Notably, the meridional transport is absent. Since the timescales for the meridional transport are comparable to some of the vertical transport timescales (such as gravitational settling and eddy diffusion), then our models lose a little fidelity, especially when we consider a range of latitudinal profiles. The main focus of this work, however, is an exploration of the consequences of the various processes at work in the Venus clouds, so the comparison to the nominal case is more relevant than a comparison to observation, in many cases.

### Atmospheric profiles

The atmospheric profiles defined by the Venus International Reference Atmosphere (VIRA) (Seiff et al. 1985) are assumed in all simulations. The atmospheric structure is held constant in all simulations presented here. Because we have not yet incorporated a dependence upon photon availability in the photochemical calculations, we simulate only the equatorial profile for the later simulations that include photochemistry.

In all cases, vertical transport of vapors is controlled solely by eddy diffusion. We assume the eddy diffusion profile defined by James et al. (1997) and McGouldrick and Toon (2007) for the altitudes 40–60 km. That eddy diffusion profile consisted of a region from 40 to 48 km with a value of 10 m$^2$/s, and a region from 57 to 60 km having a value of 2 m$^2$/s. Between these altitudes of 48 and 57 km, the eddy diffusion coefficient followed an asymmetric Gaussian that peaked at 54 km altitude with a value of 60 m$^2$/s. Note that this peak in the eddy diffusion coefficient drives much of the evolution of the cloud in these simulations. For simulations extending to 80 km, we assume a constant eddy diffusion coefficient of 2 m$^2$/s from 60 to 80 km.

### Vapor boundary and initial conditions

All simulations begin with zero particles within the model domain. In all simulations presented here, I define a water vapor boundary condition of 30 ppmv at 40 km altitude (Pollack et al. 1993). Similarly, a sulfuric acid boundary condition of 10 ppmv is set at 40 km altitude Kolodner and Steffes (1998), except in a subset of simulations in which this boundary condition is reduced to 5 ppmv. The top boundary condition for vapors assumes a constant mixing ratio above the top boundary. For sulfuric acid, that means the mixing ratio defined by 99% relative humidity, assuming the temperature and water vapor concentration extrapolated across the top boundary at the initiation of the simulation. The water vapor initial profile is assumed to be linear from 40 (30 ppmv) to 60 km (10 ppmv) and constant above 60 km. The sulfuric acid vapor initial profile is assumed to be 99% of the vapor pressure of a flat surface, until the mixing ratio exceeds the 40 km boundary condition, below which altitude, the mixing ratio is constant at that boundary value. For simulations with a top model boundary of 60 km, the top boundary condition for sulfuric acid vapor and water vapor is set to zero flux.

### Particle boundary and initial conditions

A population of involatile particles is assumed to exist below the model boundary at 40 km. The size distribution is defined by the Mode 1 particles at 45 km, as determined by LCPS observations (Knollenberg and Hunten 1980). For the sake of calculating critical supersaturations for droplet activation from a balance of the Köhler effect and solubility, these CCN are assumed to dissolve as though they were ammonium sulfate [($\hbox {NH}_4$)$_2\hbox {SO}_4$] in water.

For simulations having a top boundary of 60 km, the following changes are made. A population of sulfuric acid droplets is assumed to exist above the model boundary. These droplets are assumed to have a distribution consistent with the Mode 2 and Mode 1 particles observed at 58 km, as determined from the LCPS observations (see Table 1). The interested reader is invited to look ahead to Fig. 5, where the $+$ symbols indicate the LCPS size distributions. Those from 60 and 45 km are used as boundary conditions. However, for simulations with an upper boundary of 80 km, no particles are assumed to exist above the model top boundary. Though recent observations by SOIR on Venus Express suggest that particles may exist at altitudes above 80 km (Wilquet et al. 2012), and possibly above 90 km (Wilquet et al. 2009), their total mass hence total contribution to the cloud structure will be minimal, so it is expected that this simplification will have a minimal effect on our results.

### Photochemistry

For simulations that extend to a top model domain of 80 km, it is necessary to include at least a simplified description of photochemical production rates. Rather than simultaneously solve hundreds of chemical equations, we choose to simplify the chemical processes to the particular drivers of the microphysics. Specifically, following Yung and DeMore (1982), we assume that the net reaction producing sulfuric acid vapor in the upper atmosphere of Venus can be expressed as follows:

1 $$\begin{aligned} \hbox {H}_2\hbox {O} + \hbox {O} + \hbox {SO}_2 \rightarrow \hbox {H}_2\hbox {SO}_4 \end{aligned}$$

It is assumed that sufficient oxygen atoms are always present, supplied by either $\hbox {CO}_2$, or CO, for example, and that the reaction is generally either water or photon-limited. To simplify the chemistry, we assume that the $\hbox {SO}_2$ abundance is constant with time. While this is not strictly true, and other work suggests that the $\hbox {SO}_2$ abundance and $\hbox {H}_2$O abundance are closely linked (Parkinson et al. 2015), in our initial attempts at simulating both the microphysics and the chemistry, when we considered the role of $\hbox {SO}_2$ in the production of $\hbox {H}_2\hbox {SO}_4$, we found that the model became untenably unstable, so we deferred further work on that aspect of the problem until a later date. We assume a photochemical production rate as defined by Esposito et al. (1979) from the vertical mass flux required by the existence of the upper cloud and refined by later calculations by Krasnopolsky and Pollack (1994). That is, we assume sulfuric acid production and water vapor loss rates to be defined as:

2 $$\begin{aligned} \Phi _{\hbox {H}_2\hbox {SO}_4}&= 2\times 10^{11}\frac{\hbox {molecules}}{\hbox {cm}^2\,\hbox {s}}\cdot (\hbox {q}_{\hbox {H}_2\hbox {O}}/10 \,\hbox {ppmv}) \cdot \cos \phi \end{aligned}$$

3 $$\begin{aligned} \Phi _{\hbox {H}_2\hbox {O}}&= -\Phi _{\hbox {H}_2\hbox {SO}_4} \end{aligned}$$

The $q_{\hbox {H}_2\hbox {O}}/10\,\hbox {ppmv}$ term ensures a scaling of the photochemistry to be the nominal rate at a water vapor concentration of 10 ppmv at 60–62 km and lower production at lower concentrations. The $\cos \phi$ term represents the scaling according to latitude ($\phi$), such that the photochemical production rate vanishes at the pole. The latitude used in each profile is the mean latitude of the range defined by the VIRA profile. The photochemical production and loss are assumed to occur completely within the 60–62 km layer of the model.

### Binary homogeneous nucleation

The production of new cloud droplets can proceed via activation of existing cloud condensation nuclei (CCN) or via binary homogeneous nucleation of water and sulfuric acid molecules.

In our previous work, we assumed that a known population of particles observed by LCPS and other descent probes could serve as CCN for activating sulfuric acid cloud droplets. These small particles were observed at altitudes well below that which sulfuric acid may stably exist in the liquid phase, so it can be safely assumed that they are not sulfuric acid, but can perhaps serve as activation sites for sulfuric acid. For our simulations that extend above 60 km, however, there is no solid evidence for the existence of involatile particles. Theoretical chemical studies suggest that a population of sulfur allotropes should exist (Yung and DeMore 1982; Toon et al. 1982), but it is unclear how suitable as CCN these sulfur particles might be, given their immiscibility in liquid sulfuric acid (Young 1983). On the other hand, other researchers have suggested that micrometeorites could serve as CCN (Gao et al. 2014), but their numbers and properties can only be assumed, as no such population of particles has ever been observed and characterized in the Venus atmosphere.

Alternately, the droplets at these altitudes might form via binary homogeneous nucleation. Once a sufficient number of sulfuric acid and water molecules obtain a large enough number density via random motions, then van der Waals forces can take over and lead to the nucleation of a stable germ (collection of molecules). Because extremely high supersaturations are required to reach this state, once a germ is formed, it has the potential to rapidly increase in size via diffusional growth. Because the processes that drive nucleation are so rapid, it is necessary to parameterize the activation process in a microphysical model. Here, we use the parameterization by Vehkamäki et al. (2002). This parameterization is not valid for the entire range of conditions expected in the Venus clouds. It is constrained according to the following ranges in number of acid molecules ($N_{\mathrm{acid}}$), temperature (*T*), and relative humidity of water vapor ($\mathrm{rh}$):

$$\begin{aligned} N_{\mathrm{acid}}\,\in\, & [10^4, 10^{12}]\,\hbox {cm}^{-3}\\ T\,\in\, & [230, 305]\,\hbox {K}\\ \hbox {rh}_{\mathrm{water}}\,\in\, & [0.01\%, 100\%] \end{aligned}$$

**Fig. 1:**
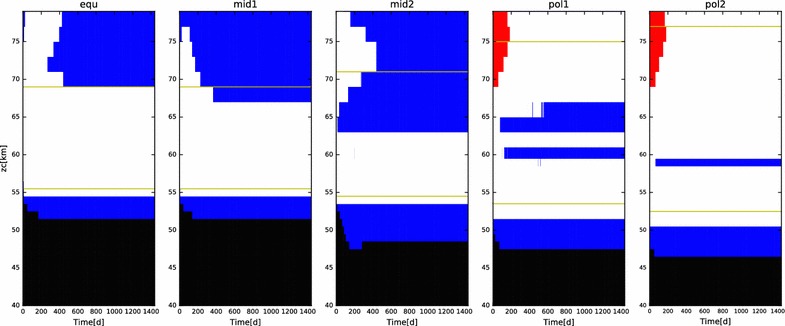
Binary homogeneous nucleation ‘truth table’ for the five latitudes at the start of a typical simulation. Color scheme is as follows. Red: $N_{\mathrm{acid}}$ out of bounds; blue: $\hbox {rh}$ out of bounds; black: both $N_{\mathrm{acid}}$ and $\hbox {rh}$ out of bounds; white: neither out of bounds; gold lines: altitudes bounded by temperature condition. In summary: only white squares between the gold lines are capable of supporting binary homogeneous nucleation

A figure depicting a truth table that indicates where the parameterization is valid for the initial conditions of the model at all latitudes is shown in Fig. 1. Note that the regions where this parameterization is invalid tend to have sufficient numbers of existing droplets and/or CCN to take up the sulfuric acid vapor so that the supersaturations never achieve magnitudes large enough to trigger droplet activation. In cases where there are insufficient CCN to generate new particles, and where the binary homogeneous nucleation scheme is not valid (for example, around 80 km at all latitudes in our models), we assume that no nucleation can occur.

## Results

### Condensational cloud models

In order to be able to distinguish between the effects of these changes on the condensational cloud versus the coupled photochemical and condensational clouds, we first perform a series of simulations on a model with a domain from 40 to 60 km, in which the photochemical upper cloud is treated as a boundary condition of particles at the top of the model domain.

#### Nominal

This simulation is performed in order to verify that the updates made to the model since its last published use have not significantly altered the characteristics of the simulated cloud. The intention is to demonstrate the consistency between the new and the old simulations, hence to lend additional credence to the current results. We perform these simulations for the five latitude regions specified in the VIRA model. As a shorthand, we call the 0°–30° profile, ‘equatorial,’ the 30°–45° and 45°−60° profiles ‘mid-latitude’ (with indices 1 and 2, respectively), and the 60°–75° and 75°–90° profiles ‘polar’ (also indexed). The equatorial profile in these simulations is the one most fairly compared to the previous simulations by McGouldrick and Toon (2007).

**Fig. 2:**
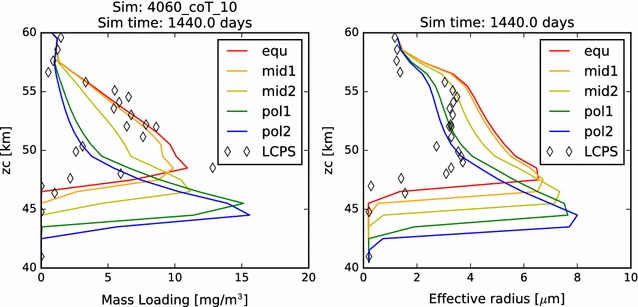
In this figure and all similar subsequent figures, the colors of the curves represent the five latitudinal profiles simulated. Red: 0°–30°, orange: 30°–45°, yellow: 45°–60°, teal/cyan: 60°–75°, blue: 75°–90°. The LCPS observations are calculated using the three modes of particles defined by Knollenberg and Hunten (1980) and are designated in all cases as diamonds. One LCPS data point at about 30 mg/m^3^ and 48 km is not shown in the mass loading figure, being off-scale, right. Here, we plot (left) the mass loading and (right) effective radius as a function of altitude for our nominal 40–60 km simulation. That is, all microphysical calculations are included, and the photochemical cloud is treated as a boundary condition at 60 km

In Fig. 2, we compare the simulated mass loading and effective radius profiles at steady state from each of our five latitudinal profiles with the same quantities derived from the in situ measurements carried out by the Pioneer Venus Large probe Cloud Particle size Spectrometer (LCPS). Knollenberg and Hunten (1980) fit three size distribution curves (associated with three distinct modes of particle populations) to their data, and it is the particle population defined by these up to three size distributions (because at some altitudes, fewer than three distinct particle size modes were required to fit the measurements) that I plot in each figure where LCPS observations are noted. Following Pollack et al. (1980), we reduce the total number concentration of the Mode 3 particles at every altitude by a factor of 3, in order to improve the consistency between the particle measurements made by LCPS and the backscattering observations made by the Pioneer Venus nephelometer.

It is important to note that the LCPS profile merely represents a single profile (at 3°S) of particle size characteristics from a single instrument falling through the Venus atmosphere. Though these nearly 40-year-old measurements represent the highest resolution in situ measurements of the Venus clouds even today, we should not afford too much meaning to them. Nephelometers, remote sensing, and the slightly lower-resolution particle size spectrometers on the VeGa landers all indicate that the size distribution and number concentration of the aerosol in the Venus clouds are both highly variable in space and time. These measurements also show that the ‘middle’ cloud deck between about 50 and 57 km is a bit more stable over time, when compared with the much more flighty ‘lower’ cloud deck. Hence by comparing our simulations with these data, we are comparing the best available ground truth at our disposal. We furthermore are more concerned with the order of magnitude agreement than the details; consistency between simulation and observation in the 50–57 km region is taken to be more of an indicator of success than the highly variable 48–50 km region, and the somewhat undersampled region above 60 km.

The mid-latitude and equatorial profiles closely match the mass loading of the LCPS observations throughout the condensational cloud. However, each of these lacks the sharp increase in cloud mass loading observed in the LCPS observations as the ‘lower cloud’ between the altitudes of about 48 and 50 km. This morphology of the vertical profile represented by the observed lower cloud, however, is closely matched by the two polar profiles, if not quite in magnitude, falling a factor of two short, although this sharp increase in mass loading occurs at much lower altitudes (~ 45 km) than observed by LCPS (~ 49 km), due to the deeper location of the cloud base at those polar latitudes. Since the 40 km boundary condition on sulfuric acid is identical across all latitudes simulated here, the lower cloud base at the polar latitudes is due entirely to the cooler temperature profile within the 40–60 km region of the Venus atmosphere.

Also in Fig. 2, we compare the effective—*i.e.*, area-weighted average—radius of the aerosols in our simulations to that derived from LCPS data. In each case, there is some agreement with the LCPS profiles. Typical effective radii through the simulated middle cloud (between about 50 and 57 km) are between about 2.5–3.0 μm among the five simulations, while the radii calculated from the distributions used to fit the LCPS measurements were just under 4.0 μm. Each profile demonstrates an increase in effective radius with proximity to the cloud base. This is partly the result of evaporation of cloud particles, which tends to remove the smallest particles entirely, while reducing only slightly the typical sizes of the larger particles. It is also the result of a steady growth of cloud aerosol that results from the increasing vapor pressure (hence, available sulfuric acid vapor) with depth.

**Fig. 3:**
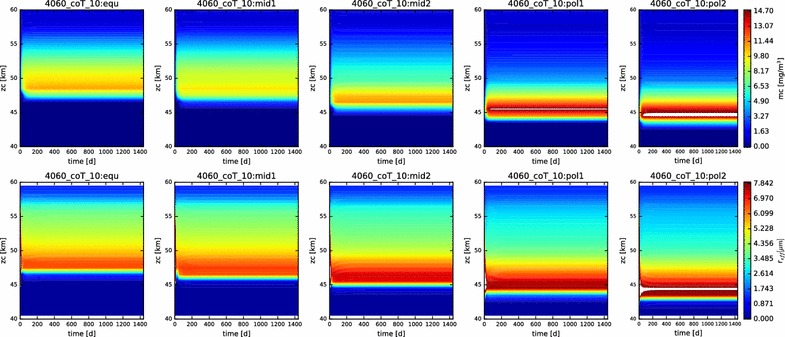
Mass loading (top row) and effective radius (bottom row) for each of the five latitude profiles simulated here (equatorial on left, polar on right). Time (in Earth days) is on the x-axis, altitude on the y-axis

In Fig. 3, we show contour plots of the mass loading and effective radius of the aerosols as a function of time and altitude for each of the five latitudes profiles simulated. The simulations are all evidently in steady state, and they reached this state (at least for the particles) very soon after the beginning of the simulation (in the first year of simulated time). This suite of models is presented to demonstrate the reproduction of the vertical behavior of the condensational clouds with latitude, namely the descent of the bulk of the cloud to deeper altitudes at higher latitudes, and the increase in effective radius with increasing latitudes.

However, despite being consistent with the accepted variations in the vertical structure of the atmosphere, the deeper cloud base found in these simulations is consistent with neither remote sensing retrievals of cloud base (Barstow et al. 2012), nor in situ measurements from the north probe of Pioneer Venus (Ragent and Blamont 1980). The latter disagreement could be simply small number statistics, given the inherent variability of the Venus condensational cloud, while the former disagreement could be the result of measurement and retrieval uncertainties at low latitude, due to the observational constraints from VIRTIS on Venus Express, which in mapping mode viewed equatorial latitudes only at very high emission angles.

In Table 3, we compile the column abundance of the aerosol for each latitude for each set of simulations in this section. We also include in the table lines for the column-integrated LCPS observations, for each of the three cases: nominal [reduce LCPS by a factor of 3, as per Pollack et al. (1980)], absent (delete all mass contributions from Mode 3), and full [use the unaltered Knollenberg and Hunten (1980) profiles]. All of the vertical profiles in the nominal case compare quite favorably with the nominal in situ measurements. The polar profiles tend to achieve somewhat larger integrated cloud mass than LCPS, but only by a few percent. Note, however, that observations by Jenkins et al. (2002) suggest a decrease in sulfuric acid mixing ratio at the cloud base from polar to equatorial latitudes. Changing this boundary condition also might significantly affect the total mass of the simulated condensational cloud in the polar latitudes. We explore this possibility shortly, but one can see in Table 3 that a reduction in the 40 km sulfuric acid vapor boundary condition by a factor of two (simulation ‘qa5’) reduces the column abundance by about 30%.

**Fig. 4:**
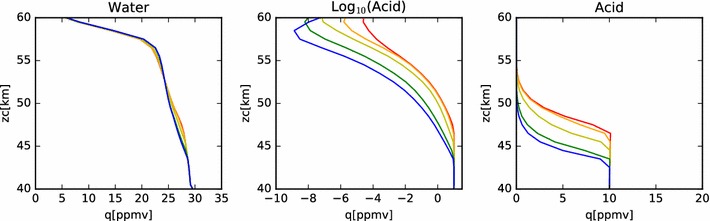
Mixing ratios of water and acid vapor in the nominal model. The several latitude profiles are identified by color, using the same scheme as in Fig. 2

In Fig. 4, we show the mixing ratios of this nominal model at steady state. In the right-hand plot, we see that, as expected, the mixing ratio of the sulfuric acid is well mixed from the bottom boundary to the cloud base at each latitude. The mixing ratio then follows the sulfuric acid vapor pressure curve until the top boundary. Because the vapor pressure drops so quickly with temperature (hence, altitude), we show the same data in a log plot in the middle panel. On the left-hand panel, we show the water vapor mixing ratio profile. Here we see a steady decrease from 30 ppmv at the bottom boundary condition, to a little more than 20 ppmv near the top of the condensational cloud at 57 km. Above this, the profile drops sharply to the 1 ppmv value set as the upper boundary condition. The steady decrease through the region of the condensational cloud is the result of the interaction between the cloud droplets and the local water vapor environment. The equilibrium weight percent of the sulfuric acid to water ratio in the droplets is determined by the ambient temperature and the water vapor concentration. Hence, as particles fall from the top of the model domain (where temperatures are cooler and the equilibrium acid mass fraction is lower (*i.e.*,  the water mass fraction is higher) in the aerosol, then water is liberated. The water vapor mixing ratio indicates the transport of water from the cloud droplets introduced at the upper model boundary into the cloud domain. The equilibrium weight percent of acid decreases from about 80% near the top of the model domain to over 90% near the cloud base. Thus, as droplets fall through the cloud, they tend to lose water in order to maintain equilibrium with their surroundings. Note that this implies an upward diffusion of water vapor from cloud base to cloud top. However, because the deposition region for the water vapor lies lower in altitude than the prescribed increase in eddy diffusion coefficient, this water is slow to be redistributed back to the cloud top and plays a role in the differences between the simulated cloud among the various latitude profiles.

None of our latitudinal profiles demonstrate a significant gap in the clouds, as indicated by the LCPS observations at about 57 km. However, we note that in McGouldrick and Toon (2007), this minimum in cloud mass loading was only seen when the radiative-dynamical feedback was being considered. It is possible that the actual radiative cooling of the cloud top drives not only the mixing within the condensational cloud, but also is responsible for this feature in the observed vertical distribution of the clouds.

**Fig. 5:**
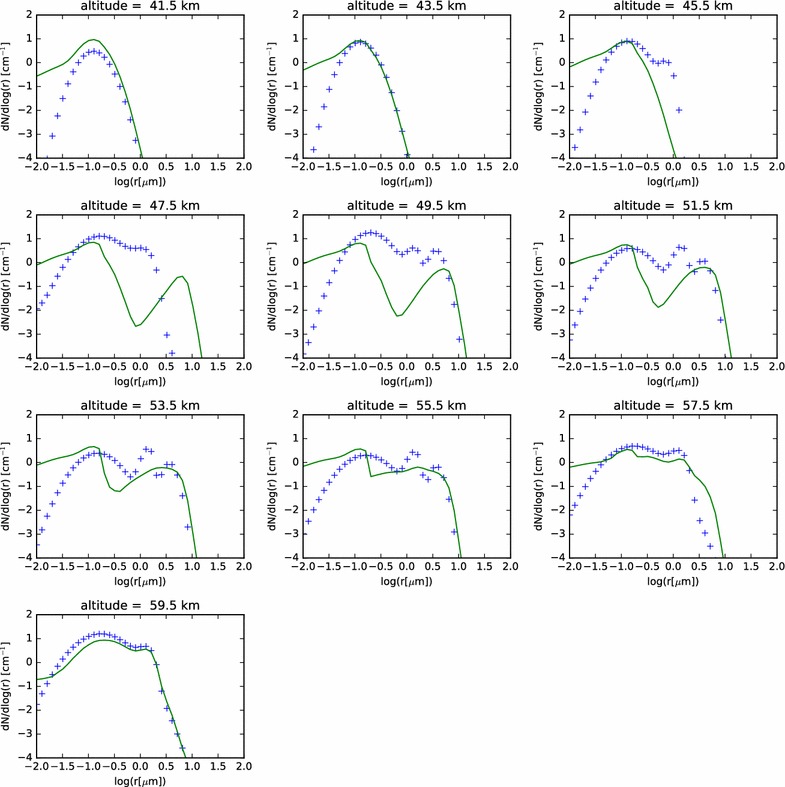
Size distribution of all particles (both involatile CCN and volatile sulfuric acid droplets) in the nominal model, at each modeled altitude, compared with the LCPS distributions ($+$)

In Fig. 5, we show the modeled aerosol size distributions compared with those derived from the in situ LCPS observational data. Since the boundary conditions are set from the LCPS data, the close match of the CCN profile to the observations in the subcloud region, and the close match of the cloud particles to LCPS near 60 km should not be surprising. However, a few items are noteworthy in these plots. First of all, at most altitudes, a mode of particles that is most consistent in size with the Mode 3 particles of Knollenberg and Hunten (1980) is seen in the sulfuric acid droplets. At the same time, a second, smaller (*i.e.*, 1.0 μm) mode of sulfuric acid droplets is not seen, with the exception of the model layers closest to the top boundary condition. Finally, the CCN involatile mode appears to do a good job of matching the observational data, except that the largest particles of Mode 1 appear to have been activated and have since grown into larger cloud particles. Some of these seeming discrepancies may be related. For instance, I have not calculated the deliquesced radius of the CCN in subsaturated conditions. If the CCN are soluble in sulfuric acid, then even at supersaturations less than the critical supersaturation, they will attract vapor molecules and swell to radii slightly larger than that of the dry CCN. While it is unlikely that this could explain the ‘missing’ Mode 2 of droplets, it would explain why the modeled CCN are actually so small (*r* < 0.3 μm) that they would not be detectable by LCPS. Note also that the typical size of the sulfuric acid droplets becomes recognizably larger than the observations only a few kilometers below the boundary condition. This may suggest either an inhibition to particle growth that we are not yet considering, or the existence of multiple sinks for the sulfuric acid. Alternately, the additional mode of particles could be a more transient phenomenon. Later in this paper, we explore these possibilities.

**Fig. 6:**
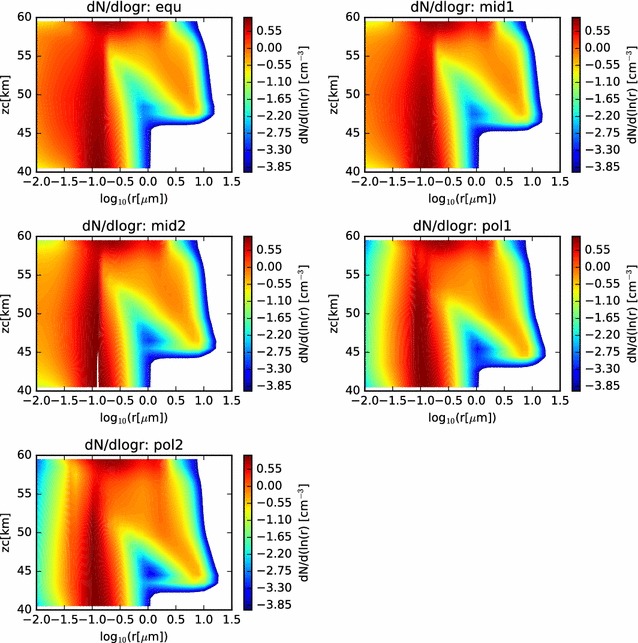
Contour plots of the size distributions previously shown as twenty separate curves in Fig. 5. Here, while comparison with LCPS results is more difficult, comparison across simulations is facilitated. In each case, both the involatile CCN and the volatile cloud droplets are considered in determining the size distributions

In Fig. 6, we show contour plots of the size distribution as a function of altitude and radius (with radius on a log scale). Here, we can see the gradual increase in radius, and narrowing of the size distribution of the larger sulfuric acid cloud droplets, as they descend through the cloud domain. The cloud base is evident to be deeper in the atmosphere at the polar latitudes, and the particle sizes have grown larger by the time they reach the cloud base. Furthermore, below cloud base, evidence of virga is seen, as the size distribution becomes more broad, reaching to smaller radii as the cloud droplets evaporate as they fall into regions of subsaturation. The scavenging of the CCN is also visible in these contour plots.

#### Suppressed coagulation model

Next, I describe the results of a set of simulations in which coagulation is strictly forbidden. This coagulation-inhibited case is not meant to represent an expected behavior in the clouds, but rather to represent an extreme end member for quantifying the effects of the efficiency of coalescence on the distribution and composition of the Venus clouds.

Returning to Table 3, we see that the aerosol column abundance of these coagulation-suppressed simulations is almost identical to the nominal case. There are slight increases in each latitude profile, from an increase of 1.76 g/m^2^ (2.5%) for the equatorial profile, to an increase of 3.62 g/m^2^ (4.8%) for the most polar profile. Note that this simulation does not permit the CCN to self-coagulate; they may only be scavenged by the droplets. And, since the number concentrations are small, Brownian coagulation is likely to be a non-factor. Gravitational collection and coalescence is a possibility, but since in our simulations the 1 μm  mode grows condensationally to become the larger mode, and the smaller mode consists of tiny CCN, this mechanism is not expected to add much mass to the condensational cloud.

**Fig. 7:**
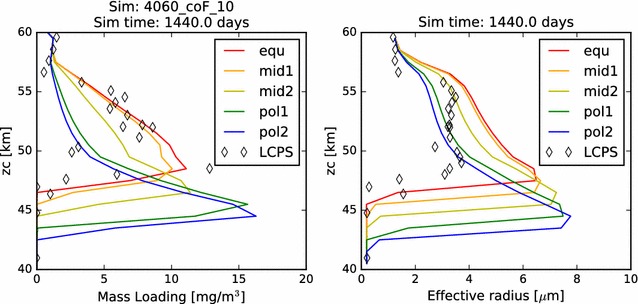
Mass loading and effective radius for the coagulation-suppressed simulations

**Fig. 8:**
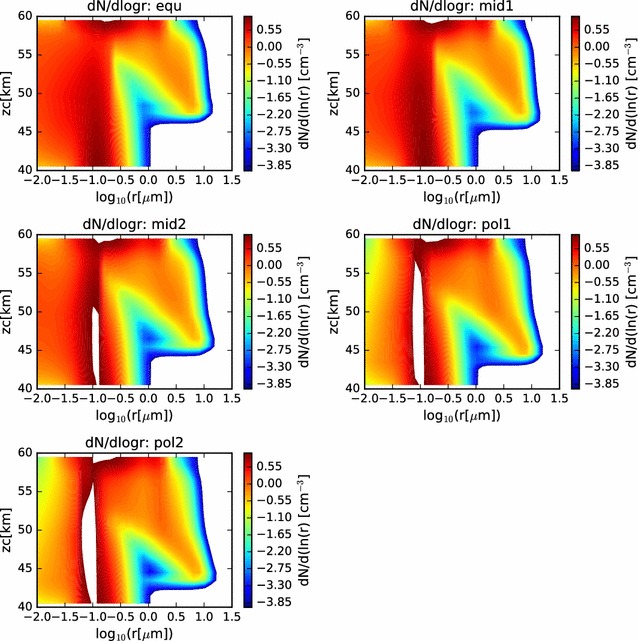
Contour plots of the particle size distributions in the coagulation-suppressed simulations

Figure 7 shows the mass loading and effective radius of the five latitude profiles in this case of suppressed coagulation of the aerosols. Since this plot differs almost imperceptibly from the nominal case, I will not discuss it further. In Fig. 8, we can see the most obvious difference between these two simulations is the much larger number of submicron-sized (in this case, involatile) particles. Namely, the suppression of coagulation has limited the magnitude of the CCN scavenging. This is most apparent in the polar latitude profiles.

#### Reduced acid vapor boundary condition

Here I describe the results of a set of simulations in which the acid vapor boundary condition is reduced by a factor of two. The reason for testing this is that the true value of the acid vapor boundary condition below the cloud base has proven rather difficult to constrain. The value of the sulfuric acid vapor mixing ratio has been most extensively measured by means of radio occultations, taking advantage of the fact that sulfuric acid absorbs radio emission at frequencies within the S-band and X-band (Kolodner and Steffes 1998; Jenkins et al. 2002). Unfortunately, at the altitude of the cloud base of Venus, the measurement errors in radio occultation profile analysis are beginning to become quite large. Previous studies by Kolodner and Steffes (1998) indicate large vertical and meridional variations, and even significant inversions in the sulfuric acid vapor mixing ratio below cloud base. Such strong observed variations must be indicative either of a tremendous amount of vertical overturning in the subcloud region, or of significant measurement uncertainties. More recent work by Oschlisniok et al. (2012) chose not to retrieve the sulfuric acid vapor mixing ratio at those deep altitudes, due to these uncertainties. In situ measurements are needed to better constrain the abundances of this and other sulfur-bearing species in the atmosphere of Venus; broad spatial and temporal coverage can then provide insight as to the relative contribution of inherent variability or measurement uncertainty in these previous measurements.

**Fig. 9:**
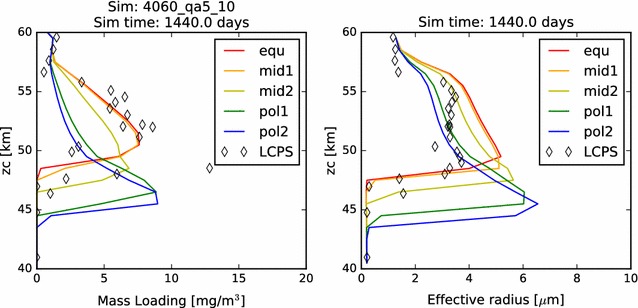
Mass loading and effective radius of reduced sulfuric acid vapor boundary conditions simulation

Figure 9 shows, as expected, the total mass loading and the typical particle sizes of the aerosols decrease in this case, while the cloud base rises slightly. These effects are most pronounced at polar latitudes. The column abundance has decreased, not by half but by about a third. The most obvious difference between this simulation and the nominal simulation is a slight reduction in the number of the largest cloud droplets, which brings about a significant reduction in overall cloud mass and in effective radius. However, also note the larger difference between the altitudes of the cloud base as a function of latitude in these reduced acid vapor boundary condition simulations.

#### Reduced upper cloud boundary condition

**Fig. 10:**
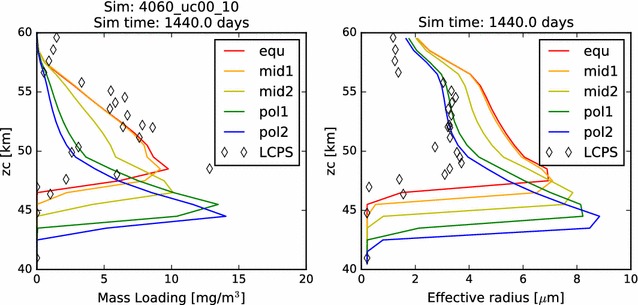
Mass loading and effective radius of suppressed upper cloud boundary simulation

In order to understand how much of an effect the upper cloud boundary condition has on the condensational cloud, we conduct a simulation in which the photochemical upper cloud source has been removed. In this simulation, the only source of sulfuric acid is the flux due to the lower model boundary condition. As shown in Fig. 10, the total mass loading of the cloud predictably is decreased in this simulation, but the magnitude is quite small, at least for the equatorial latitudes. In Table 3, we see that for the equatorial and mid-latitude profiles, the total aerosol column abundance is reduced by no more than 6%. However, the effect on the polar profiles is much more significant, exhibiting a reduction by about 20%. This might be expected, since the rate of photochemical production of sulfuric acid at polar latitudes will necessarily be smaller than that at equatorial latitudes, due to the greater average zenith angle of the Sun. That is to say that in the previous simulations, we had been overestimating the photochemical production of cloud mass contribution to the condensational cloud and that overestimation increased with latitude, because we had assumed a rate that had been defined near the equator. Now, by removing that forcing altogether, we are arriving at a simulated cloud that is more in line with what should be expected at high latitudes. In other words, the polar profiles previously simulated may have been overestimating the total mass loading, having overestimated the mass loading provided by the photochemical upper cloud at those latitudes. Hence, when we extend the model to 80 km in the simulations described in "Photochemistry models" section, we will need to consider the latitudinal variations in the photochemical production rate of sulfuric acid, if we wish to generate realistic models of the Venus cloud system.

**Fig. 11:**
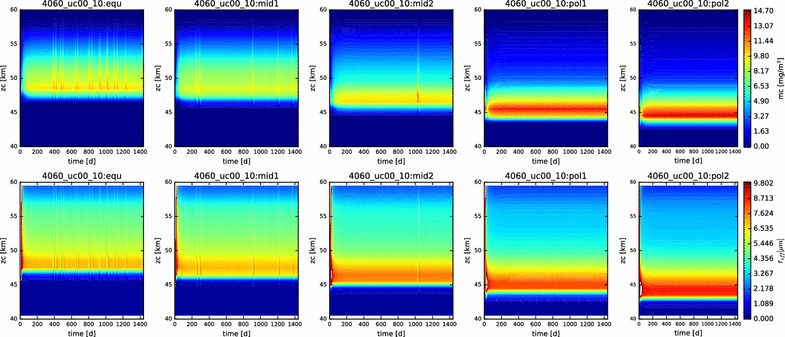
Mass loading (top row) and effective radius (bottom row) for the suppressed upper cloud boundary condition simulation

We should note the sporadic excursions of the simulated cloud from the steady state that are evident in the mass loading and effective radius contour plots (Fig. 11). These excursions produce increases in the aerosol mass column abundance on the order of about 20%, and changes of several microns in effective radius. These changes are matched with minor increases in the vapor mixing ratios, but the trend appears more consistent with the existence of bursts of nucleation or activation of the cloud particles, rather than growth processes. Due to the prescribed eddy diffusion coefficient, this disturbance propagates quickly through the cloud. However, in the current model, this will only occur if the disturbance occurs between altitudes of 48 and 57 km. Below 48 km, the lower value of the static eddy diffusion coefficient of $10\,\hbox {m}^2/\hbox {s}$ results in a longer mixing time scale which slows the mixing of the disturbance into the rest of the cloud. In a more realistic simulation, in which the vertical mixing was more directly tied to the heating and cooling rates, this change in vertical distribution of cloud mass would likely remain more localized; at least unless and until it were able to produce sufficient change in temperature to drive an episode of convective overturning. It is interesting to note that these excursions do not occur in the polar profiles, suggesting that the deeper polar condensational clouds are somewhat less affected by changes in the photochemical clouds above.

### Photochemistry models

Here, we describe the results of our simulations designed to incorporate the entire Venus cloud system, including the photochemical cloud, the condensational cloud, and the associated hazes both above and below. The only arbitrary model condition that still is applied is the stipulation of fixed boundary conditions on the vapor mixing ratios at 40 km, which are intended to provide a sink of sulfuric acid vapor and a source of water vapor, similar to what would occur if we were to simulate the thermochemistry in this domain. The photochemical production is assumed to occur only between 60 and 62 km and occurs at the rates described in "Photochemistry" section.

In the first series of simulations, we investigate the effects of the magnitude of the photochemical production rate of sulfuric acid. Numerous values are found in the literature. Esposito et al. (1979), using the in situ observations by LCPS as a guide determined the downward mass flux of the sulfuric acid aerosol at the transition between the middle and upper cloud decks. Converting this mass flux to a molecular production rate, they arrived at a sulfuric acid production rate of $2\times 10^{11}$ molecules cm$^{-2}$ s$^{-1}$. Imamura and Hashimoto (2001) reported that a photochemical production flux of $1\times 10^{12}$ molecules cm$^{-2}$ s$^{-1}$ provided the best fit to the mass loading of the lower part of the photochemical upper clouds, as determined by the in situ LCPS measurements, and so used this value in their work. Gao et al. (2014) use an intermediate rate of $6\times 10^{11}$ molecules cm$^{-2}$ s$^{-1}$, noting only that this choice of parameter provided better fits to the observed cloud that the $1\times 10^{12}$ molecules cm$^{-2}$ s$^{-1}$ used by Imamura and Hashimoto (2001). In this subsection of simulations, we first compare the results of our nominal case (using the Esposito et al. (1979) estimate) with the Gao et al. (2014) (larger by roughly a factor of three). In order to better characterize the effect of the photochemical production rate, we also consider a rate that is 1/10 of that used by Gao et al. (2014), so that we roughly bracket the nominal case above and below with rates differing by a factor of three. We do not simulate the Imamura and Hashimoto (2001) case here, expecting that the trends we see in comparing our nominal case with the larger and smaller bracketing rates will elucidate any trend in that direction.

#### Nominal model

**Fig. 12:**
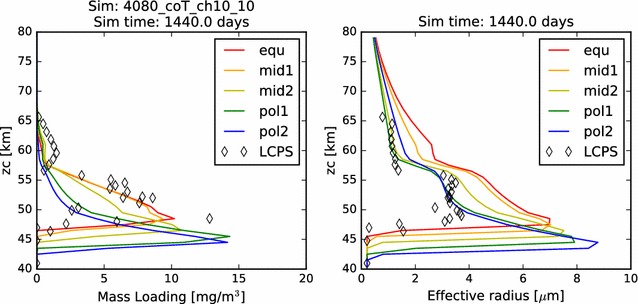
Mass loading and effective radius of the nominal model containing photochemistry, showing the full domain from 40 to 80 km

As before, we first present the mass loading and effective radius of the aerosols at the end of the four-year simulation in Fig. 12. The results of this simulation appear to most closely resemble the simulations without an upper boundary condition of supplied aerosol ("Reduced upper cloud boundary condition" section). In general, this simulation appears to underestimate the mass loading and overestimate the effective radius of the aerosol in the upper cloud region. Perhaps this is due to an overestimation of the photochemical production of acid vapor, leading to excessive supersaturations, rapid growth, and near-immediate rainout of the photochemically produced cloud aerosols. If so, then we should expect to see an improvement when we utilize the Gao et al. (2014) production rate later in this paper. On the other hand, it is possible that our focusing of the photochemical production of vapor onto the single atmospheric layer from 60 to 62 km presents a local excess in vapor concentration. In this case, our simulations would be improved by a more realistic vertical distribution of the sulfuric acid production rates. We did not simulate a vertical distribution of the photochemical production and loss rates because the clouds themselves will play a role in determining the depth to which the photolyzing photons penetrate. So, any prescribed vertical distribution of photochemical production rates will essentially assume a vertical cloud distribution. Since we are not performing rigorous radiative transfer calculations at this time, we would have had to have arbitrarily determined the penetration depth of photons for each latitude. For simplicity, we assumed the same production altitude as the equatorial profile for all of the latitude profiles. Future development of this work will include a more detailed characterization of the photochemistry.

Concentrating on the distribution of photochemical cloud mass in these simulations, we note that the profiles between 60° latitude and 75° latitude do an excellent job of matching the effective radius of the photochemical cloud measured in situ by LCPS. However, these simulations also severely underestimate the mass loading. This disagreement should be taken with a grain of salt, however, as the LCPS measurements were obtained as the large probe descended at an equatorial latitude of 3°S, so agreement with mid-latitude profiles, especially with regard to the upper cloud (where the atmospheric profiles are more significantly different), is not something to be expected, unless we have created a situation where two wrongs make a right.

**Fig. 13:**
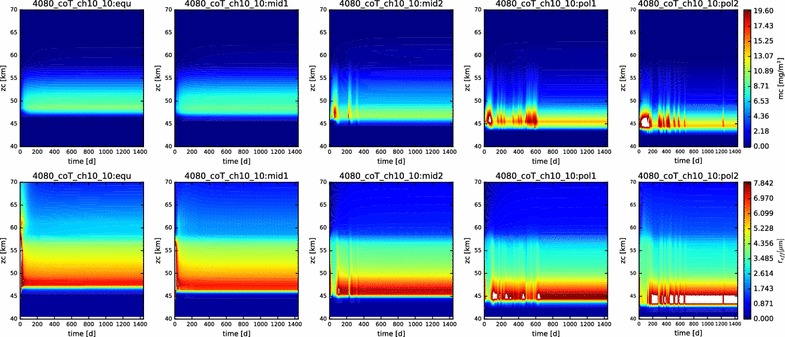
Contour plots of mass loading and effective radius for the nominal simulation containing photochemistry, displayed over the domain from 40 to 70 km

The contour plots of the mass loading and effective radius with time in this simulation (Fig. 13) demonstrate that we are at or near steady state. However, note that the more polar the latitude, the more unstable the simulated cloud is at the outset. This is perhaps to be expected, since our prescribed photochemistry applies most relevantly only at the equatorial latitudes.

In Table 4, we again compare the column abundance of cloud mass in the simulation with that seen by LCPS. In order to facilitate comparison with the previous simulations, we divide the table into two regions: 40–57 km (condensational cloud) and 57–80 km (photochemical cloud). Some caveats, however, include the recognition that since the altitudes of the cloud layers vary with latitude, a strict altitude cutoff is perhaps not the ideal way to distinguish between the two regions. However, since the bulk of the condensational cloud in each case is well below the 57 km dividing altitude, and the photochemical production region is fixed to 60–62 km, well above this dividing altitude, this choice of segregation altitude is a reasonable one. Also, it remains important to note again that the LCPS measurements were taken only at equatorial latitudes.

Many of the simulations did not achieve a true steady state. Rather, they tended to oscillate between two end-member quasi-steady-state profiles. Because of this, it is necessary to express the column abundance in terms of a mean and variation in Table 4. This was not done for the previous simulations because they had all reached steady states in which the day-to-day variations were minuscule compared with the mean steady-state values achieved. In Table 4, we report the mean and standard deviation of the integrated aerosol column mass abundance over the final two years of the simulation. It is assumed that any contribution to the statistical variance from the build up from zero to a quasi-steady state will be reduced by having chosen such a latter half-time window.

As mentioned above, the condensational cloud in this simulation (and indeed, all of the chemistry sensitivity test simulations, which will be discussed below) contains smaller mass loadings that are more consistent with the 40–60 km simulations that suppressed the upper cloud boundary condition. Furthermore, as might be expected, the changes in the photochemical production rates had a larger effect on the column abundance of the photochemical cloud than on the condensational clouds. This is likely a robust result, assuming that the timescales for the thermochemical destruction of sulfuric acid, and subsequent depletion of the reservoir, are likely to occur at somewhat longer timescales than the microphysical processes observed here (Krasnopolsky 2007). At this point, considering only the nominal simulation, we see that the column abundance of the photochemical clouds is underestimated in almost every latitude of every simulation. Only the 45°–60° latitude, ‘mid2’ profile exhibits a photochemical cloud column abundance that is larger than reported by the LCPS in situ measurements. However, the condensational cloud mass loading is underestimated slightly at low latitudes and overestimated at higher latitudes. Furthermore, the condensational cloud appears to be more susceptible to larger excursions from the steady-state mean at higher latitudes. The photochemical cloud, despite differences across latitude and simulation parameters, tends to maintain a 20–30% level of variability in all cases. The condensational cloud at equatorial latitudes exhibits variability at the roughly 10% level, while at higher latitudes can exhibit variability of more than 20%.

**Fig. 14:**
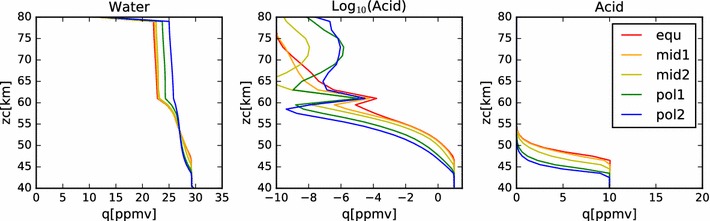
Mixing ratios of water, acid and log(acid) for nominal photochemistry simulation

In Fig. 14, we show the acid and water mixing ratios with altitude for this simulation. As with the earlier models of just the condensational cloud, the acid vapor profile is constant below the cloud base and follows the saturation vapor pressure curve within the clouds. The water vapor profile also resembles the condensational cloud case, except there is more variation with latitude at altitudes above 60 km. Note that these values of water vapor mixing ratio are all significantly larger than the $2\pm 2\,\hbox { ppmv}$ of water vapor reported by Cottini et al. (2015) above the cloud tops (while the derived cloud top altitude varied with latitude over a range from about 64 km to about 71 km). However, these simulated values of water vapor mixing ratio are consistent with the analysis of SPICAV-UV data done by Fedorova et al. (2016), who found water vapor mixing ratios varying between 4 and 11 ppmv near the equator, measured at a sampling altitude a few kilometers deeper than that sensed by VIRTIS. The spike in sulfuric acid vapor mixing ratio seen in all simulations and latitudes at 61 km is due to the photochemical production applied at that altitude. Note that the peak of this spike is smaller at higher latitude, showing the effect of the decrease in photochemical production rates as a function of solar zenith angle. The variability above 65 km is due to variations in the vapor pressure of sulfuric acid in response to changes in the temperature and water vapor concentration. Another contributing factor is the validity of the binary homogeneous nucleation parameterization. At locations where the defining parameters stray outside their bounds, the acid vapor cannot be converted into new particles by the model, so the vapor will build up until a favorable place in the parameter space is reached. Future work intends to make use of an improved binary homogeneous nucleation scheme currently under development (Määttänen, personal communication). Note, however, that while there are orders of magnitude variation in the acid vapor mixing ratio, in all cases these values are smaller than $10^{-6}\,\hbox {ppmv}$; that is, less than the parts per trillion level (about 10^6^ molecules/cm^3^ at 70 km).

**Fig. 15:**
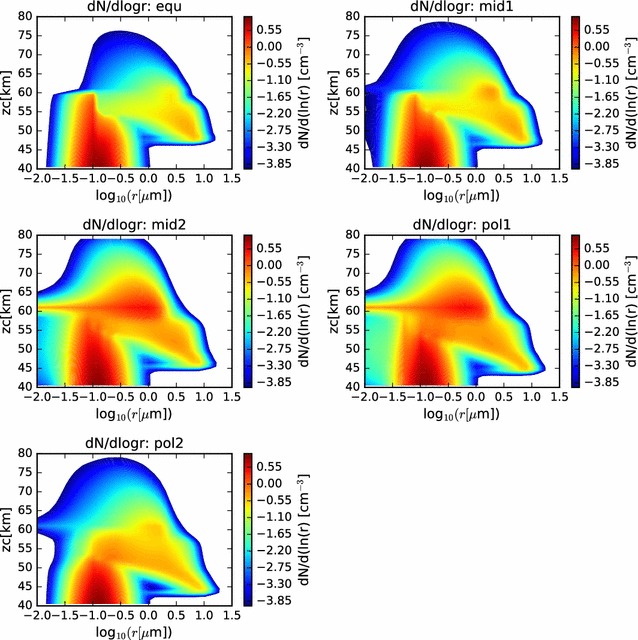
Contour plot of particle size distributions for the nominal photochemistry simulation, as functions of radius and altitude, for each simulated latitude profile

In Fig. 15, we show the particle size distributions at the end of the simulation as functions of radius and altitude. We see many similarities to the nominal models of the condensational cloud, especially in the 40–60 km altitude range of each latitude profile. At the 60–km altitude layer of the model, we see the effect of the photochemical production most evidently in the three profiles at higher latitudes (‘mid2’, ‘pol1’, and ‘pol2’). In those three profiles, a long trail of enhanced particle numbers extending to very small radii at 61 km is seen. This is evidence of the binary homogeneous nucleation, followed by condensational growth, occurring in the simulation. When supersaturations reach sufficiently high levels, and if there is an absence of suitable condensation nuclei, then a large number of very small droplets, called germs, are formed. These germs have radii of only a few nm, but are very numerous. These grow very quickly by condensation and Brownian coagulation nearly to micron sizes. Any particles that diffuse to higher altitudes can continue to grow at their leisure, as sulfuric acid gradually builds, having been produced at the photochemical production altitude and diffused upward. Eventually, particles become large enough to sediment into the condensational cloud, where they can experience further condensational growth, as seen in the earlier condensational cloud simulations.

In the ‘equ’ and ‘mid1’ profiles, we do not see obvious evidence for the generation of numerous small droplets. This is possibly because sufficient CCN have reached to this altitude in these simulations, permitting activation of sulfuric acid aerosols, hence short-circuiting the binary homogeneous nucleation step. This suggests that the presence of suitable CCN can have a significant effect on the structure of the upper part of the Venus clouds, including the photochemical cloud. Note that previous work by James et al. (1997) suggested that the multimodal structure observed by LCPS could only be obtained when solubility of the CCN was taken into consideration. However, Toon et al. (1984) pointed out that the most likely candidate for the involatile CCN observed by LCPS was sulfur allotropes, which Young (1983) noted are not miscible in sulfuric acid. Consequently, the Mode 1 particles cannot serve as CCN for the condensation of small sulfuric acid droplets if they are sulfur allotropes. Since our simulations more closely match the observations only when binary homogeneous nucleation is evident and provide a less good match when heterogeneous nucleation is occurring near the cloud tops, this may suggest that the structure of the Venus clouds, especially the photochemical clouds, can only be obtained through the consideration of **insoluble** involatile particles. Thus, either the Mode 1 particles are not soluble in sulfuric acid and cannot be considered to reduce the vapor pressure via the solute effect in the condensation of sulfuric acid in the condensational clouds, or the processes of aerosol activation and scavenging by coagulation must remove all of the Mode 1 involatile particles before they can reach the photochemical cloud. In this scenario, the Mode 1 particles are then understood to be a population of sulfur allotropes or other species that is immiscible in sulfuric acid. These particles will be ubiquitous throughout the clouds, and below. The Mode 2 particles are the products of photochemical production of sulfuric acid, binary homogeneous nucleation and subsequent condensational and coalescent growth. Any particles that could be considered Mode 3 would then simply be the large size wing of the size distribution in these simulations.

**Table 1:** Summary of nominal models

Altitude (km)	Condition	Value
40	Acid vapor	10 ppmv
40	Water vapor	30 ppmv
40	CCN (involatile)	(Lognormal)* N*_tot_ = 200 cm^3^, $r_m=0.125\,\upmu \hbox {m},\, \ln {\sigma }=2.16$
40	Droplets	0
60	Acid vapor	Constant supersaturation across boundary
60	Water vapor	1 ppmv
60	CCN	0
60	Droplets	(Lognormal) $N_{\mathrm{tot}}=600\,\hbox {cm}^{-3}$ $0.175\,\upmu \hbox {m}$, $\ln {\sigma }=2.16$, (Normal) $N_{\mathrm{tot}}$=80 cm$^{-3}$ $1.14\,\upmu \hbox {m}$, $\sigma =1.4\,\upmu \hbox {m}$
80	Acid vapor	Constant supersaturation
80	Water vapor	Constant mixing ratio
80	Particles	0
All	T, P, $\rho$	VIRA at five latitudes
40–48	$K_{\mathrm{diff}}$	10 m$^2$/s
48–54	$K_{\mathrm{diff}}$	Gaussian [see James et al. (1997)]
54–57	$K_{\mathrm{diff}}$	Gaussian [see James et al. (1997)]
57–80	$K_{\mathrm{diff}}$	2 m$^2$/s

**Table 2:** Summary of simulations performed

Upper bound (km)	Sim name	Description
60	4060_coT_10	Nominal model
60	4060_coF_10	Coagulation completely suppressed
60	4060_qa5_10	Acid vapor 40 km boundary condition: 5 ppmv
60	4060_uc00_10	Particle upper boundary condition suppressed
80	4080_coT_ch10_10	Nominal
80	4080_coT_ch11_10b	Increased photochemistry rates
80	4080_coT_ch12_10	Decreased photochemistry rates
80	4080_coF_ch10_10	Suppress all coagulation
80	4080_coV_ch10_10	Suppress coagulation when *T* < 283 K

**Table 3:** 40–60 km column abundance (all units g/m$^2$)

Latitude	Simulation			
	Nom	coF	qa5	uc00
equ	71.3	73.0	44.9	70.5
mid1	71.6	73.2	46.0	69.0
mid2	70.9	72.6	46.8	70.9
pol1	74.6	77.3	48.7	63.1
pol2	75.4	79.0	47.9	61.4
LCPS, Mode 3 = 0	9.00 g/m^2^			
LCPS, Mode 3 = 1/3	69.0 g/m^2^			
LCPS, Mode 3 = 1	189.0 g/m^2^			

**Table 4:** Column abundance for the 40–80 km simulations (all units g/m^2^)

Model and altitude		Latitude				
		equ	mid1	mid2	pol1	pol2
Nom	CC	$60.4\pm 6.74$	$62.8\pm 6.79$	$67.8\pm 8.28$	$74.1\pm 13.7$	$71.1\pm 16.9$
Nom	PC	$2.10\pm 0.482$	$2.55\pm 0.618$	$3.83\pm 0.791$	$2.74\pm 0.594$	$0.985\pm 0.175$
ch11	CC	$61.4\pm 6.95$	–	–	–	–
ch11	PC	$2.92\pm 0.655$	–	–	–	–
ch12	CC	$59.8\pm 6.65$	$61.8\pm 6.89$	$65.8\pm 7.98$	$75.0\pm 20.1$	$95.1\pm 27.3$
ch12	PC	$1.48\pm 0.323$	$1.50\pm 0.315$	$2.09\pm 0.384$	$1.52\pm 0.293$	$0.848\pm 0.273$
coV	CC	$60.5\pm 6.69$	$63.0\pm 6.77$	$130.\pm 18.3$	$166.\pm 19.8$	$98.5\pm 20.3$
coV	PC	$2.13\pm 0.491$	$2.61\pm 0.642$	$12.7\pm 3.23$	$7.57\pm 1.70$	$1.21\pm 0.280$
coF	CC	$61.5\pm 6.87$	–	–	–	–
coF	PC	$2.21\pm 0.516$	–	–	–	–
LCPS	CC	69.0 g/m^2^				
LCPS	PC	8.11 g/m^2^				

*CC* Condensational cloud (40–57 km ), *PC* Photochemical Cloud (57–80 km)

#### Enhanced photochemical production rates

**Fig. 16:**
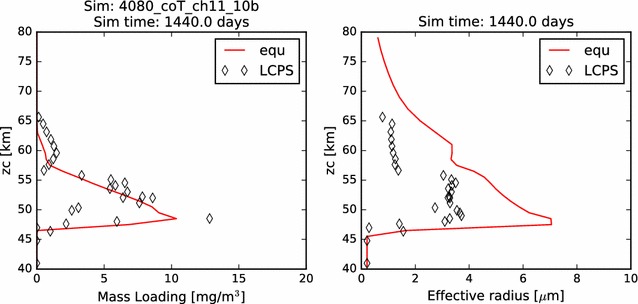
Mass loading and effective radius for the enhanced photochemical rate simulation, equatorial profile only

Here we present the results of simulations that, similar to Gao et al. (2014), utilize a photochemical production rate that is three times larger than that predicted by Esposito et al. (1979). Unfortunately, due to difficulties in avoiding non-physical results (such as negative gas or particle concentrations) when simulating the higher latitude profiles in this simulation, we can only present the equatorial profile here. In most cases, these non-physical results are caused by excessive droplet activation and excessive acid vapor supersaturation produced by a ‘ringing’ of the model as it evolves from an initial to a steady state. This early model instability can be reduced by instituting a more realistic altitude distribution of the photochemical production of sulfuric acid, or the introduction of additional sulfur chemistry to smooth away such spikes in vapor concentrations. In Fig. 16, we show the mass loading and effective radius for this simulation. Comparing to the equatorial profile of Fig. 12, we see that the effective radius of the particles in the photochemical production region is somewhat larger than those from the nominal simulation, by about 0.5–1.0 μm. However, the mass loading appears to be comparable between the two simulations at these altitudes. This can also be checked by comparison of the column abundance in Table 4, which shows that the column abundance above 57 km of this simulation is larger than the nominal by about 30%.

**Fig. 17:**
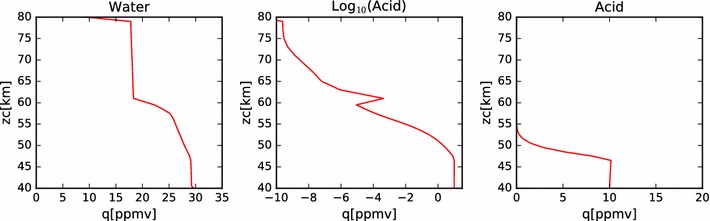
Water and acid mixing ratios for the enhanced photochemical rate simulation; equatorial profile only

The plot of the mixing ratios from this simulation (Fig. 17) indicates another consequence of the more effective photochemical production rates. The steady-state water vapor mixing ratio about the photochemical production altitude is around 18 ppmv, noticeably smaller than the 22 ppmv observed in the nominal case. Even still, this is a significantly larger value than would be expected from the Cottini et al. (2015) observations, but somewhat more consistent with the observations by Fedorova et al. (2016). This result indicates that a more efficient removal of the water vapor than is considered here will be necessary to obtain agreement with the observations in terms of water vapor abundance.

#### Diminished photochemical production rates

**Fig. 18:**
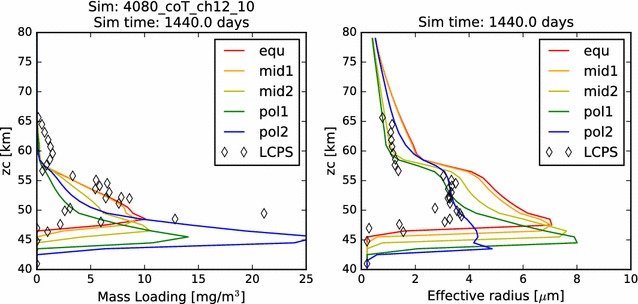
Mass loading and effective radius for the diminished photochemistry rate simulations

Consistent with the results of the simulations in which the mass loading and effective radius were found to increase in response to an increase in photochemical production rates, this simulation in which the photochemical production rate was decreased results in decreases of photochemical cloud mass and effective radius. In fact, unlike the previous two simulations, there is no obvious signature of the upper cloud in terms of mass loading in Fig. 18. Comparing the column abundances in Table 4, we see that the photochemical cloud mass in this simulation is just a bit more than half that of the nominal case, for all latitude profiles except the most polar. Curiously, the condensational cloud seems unperturbed by this change in photochemistry, except for the roughly 30% increase in column abundance exhibited by the most polar profile. Furthermore, the most polar latitude profile in this case appears to more closely match the LCPS observations in both the condensational cloud and the photochemical cloud. The effective radius of the particles remains smaller than 4 μm until very near to cloud base, where there is a large increase in particle number and mass, but only a slight increase in particle effective radius. It is worth noting here again, however, that we should be wary of drawing conclusions from matches between the observed data at equatorial latitudes and the simulations which are at polar latitudes, especially since these are one-dimensional simulations which inhibit any contributions due to large-scale dynamics. In this case, however, the comparison may be telling us something useful about the photochemistry. For example, perhaps a smaller photochemical production rate will be more consistent with the mass loading and particle distributions in the photochemical cloud. Or, perhaps the activation and nucleation of new particles, or their growth to larger sizes is less efficient than the current suite of models assumes.

#### Coagulation effects on full domain model

Having characterized the effects of the parameters that determine the photochemical parameterization in our simulations, we can now turn to the primary focus of this paper, which is an exploration of the effects of coalescence properties on the Venus cloud system. As in the first section of results, we will compare two simulations: one in which coagulation is allowed to proceed as normal, and a second in which coagulation is forbidden to occur. Then, unlike the earlier simulations, we also consider a third case in which we allow coalescence to occur for cloud droplets only when the environmental temperature is warmer than 283 K, a typical melting point for sulfuric acid under the atmospheric conditions experienced here (McGouldrick et al. 2011). Note that the melting point of sulfuric acid is a function of the weight percent of acid in solution, which is in turn a function of both the water vapor concentration and the temperature. Hence, the temperature we are currently assuming to be the melting point will differ slightly from the true melting point over the course of the simulation, as the water vapor concentrations vary (note that we hold temperatures constant in the current simulations). However, since the vertical resolution of our simulations is no better than 1 km, and the lapse rate is nearly 10 K/km, a change of a Kelvin or two will not make a significant difference in our simulations. Only if the weight percent of acid in the droplets changes significantly (for example, down to 70%, or up to 90%) will our predicted ‘melting’ altitude be in error by more than a kilometer.

As with some of our earlier simulations, the simulation in which we completely suppressed all coagulation could not be successfully completed for all five latitude profiles. As in the other failed simulations, the driving factor was excessive droplet activation by CCN in regions of the atmosphere having relatively small concentrations of acid and water vapor, and relatively long eddy diffusion transportation timescales. This is caused by coagulation suppression inhibiting the scavenging of the CCN, rather than it having inhibited the collisional growth of the droplets themselves. It also provides additional evidence that the nature of the activation and nucleation processes of new particle formation in the clouds of Venus play a very significant role in the ultimate distribution of the clouds. This also suggests the importance of the role of the radiative-dynamical feedback in regulating the vertical mixing of the condensational cloud.

**Fig. 19:**
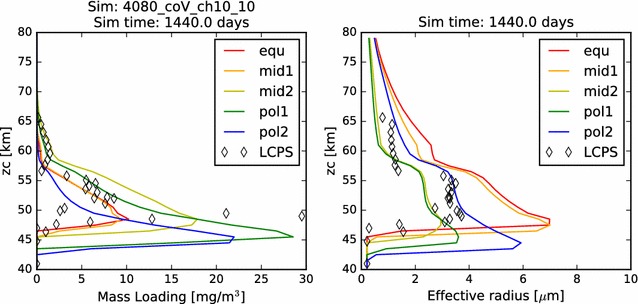
Mass loading and effective radius at the end of the temperature-variable coagulation simulation

In Fig. 19, we show the mass loading and effective radius of the aerosol in the temperature-dependent coalescence simulation. While the vertical profiles individually resemble many of those shown previously in this paper, the nature of the variations over latitude is unlike anything yet shown. On the one hand, we see that the mass loading of the photochemical clouds of the ‘mid2’ and ‘pol1’ simulations matches quite well with the LCPS observations at altitudes above 60 km. The effective radii for these profiles, however, are somewhat smaller than the observations. Second, we no longer see a monotonous progression of mass loading and effective radius from equator to pole. Certainly, the cloud base steadily decreases, but that is to be expected, since it will depend almost solely upon the temperature profiles, which have not changed. However, unlike our previous simulations, the most polar profile no longer exhibits the largest mass loading achieved in the model domain, nor the largest particle effective radius. In fact, if one considered an average of the ‘pol2’ and ‘pol1’ mass loading and effective radius profiles, one would obtain a simulated cloud that very closely matches the LCPS observational profile. Each of the simulations continues to show a sharp increase in aerosol effective radius near the cloud base, though the increase is a bit more subtle for the ‘mid2’ and ‘pol1’ profiles. This can be taken to be consistent with the LCPS observations, if tempered with the suggestion by Toon et al. (1984) that the Mode 3 particles are actually just a larger particle tail of the existing aerosol distribution. However, this would require that the collection of such a large fraction of Mode 3 particles in the lower clouds in the LCPS data was the result of some temporary dynamical phenomenon. Taken as a whole, these simulations do a very good job of matching to the LCPS observations. In any case, they do not differ enough from the observations to be able to completely rule out the plausibility of this simulated scenario.

**Fig. 20:**
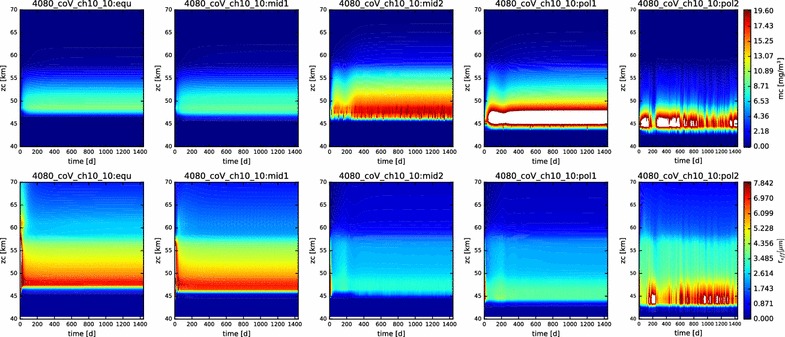
Contour plot of the mass loading and effective radius as functions of time and altitude in the temperature-variable coagulation simulation

However, Fig. 20 shows that at least part of the unexpected behavior has more to do with temporal variations than the latitudinal variations. The mid-latitude and polar profiles exhibit a much greater recurrence of significant variability in both mass loading and effective radius than previous simulations. Especially noticeable in the ‘pol2’ profile are both short time scale (a few days to weeks) and long time scale (hundreds of days) variations, especially in the lowest regions of the clouds, but also extending to higher altitudes. Such variations occurred also in previous simulations, but only as a result of a ‘ringing’ of the model as it evolved from its initial conditions to a steady state. Their persistence here throughout this simulation suggests that the temperature-variable coalescence efficiency considered here destabilizes the entire condensational cloud and could be responsible for driving some of the variation that is seen.

**Fig. 21:**
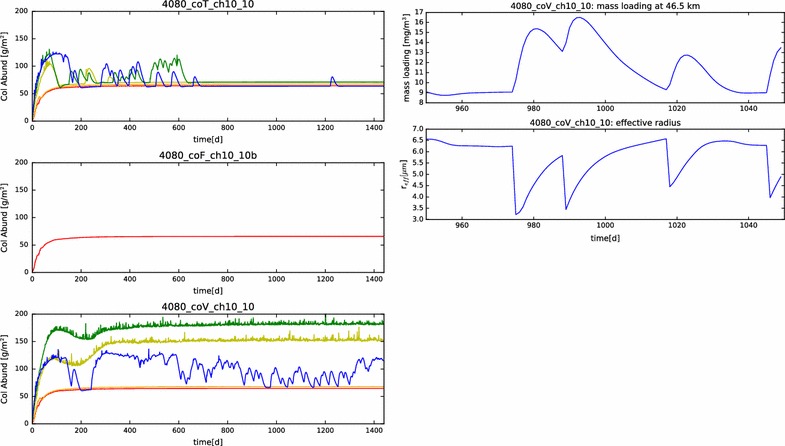
Left: The total column abundance in the microphysical model domain (40–80 km) for each latitude and coagulation condition simulated here. Right: Mass loading and effective radius at 46.5 km and ‘pol2’ profile in the temperature-dependent coagulation simulation, focusing on the time from 950 to 1050 days, showing the nature of the evolution seen in the simulation

**Fig. 22:**
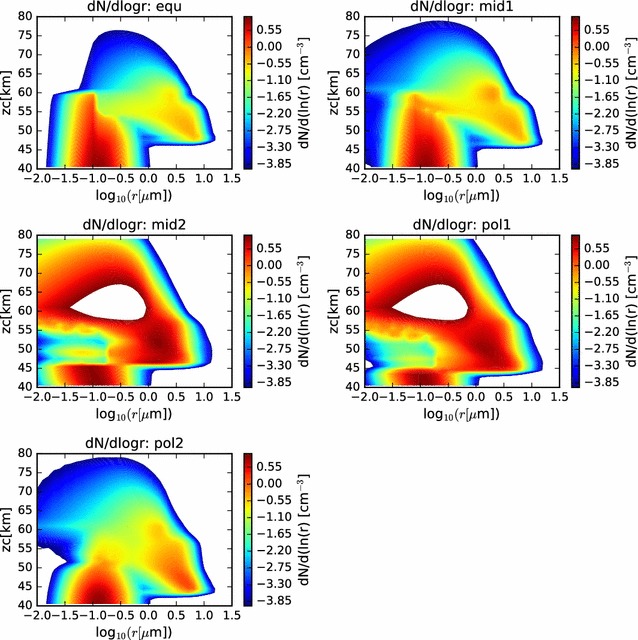
Particle size distributions at the end of the temperature-dependent coagulation simulation, shown as contour plots against radius and altitude for each of the five simulated latitude profiles

Figure 21 indicates the magnitude of the changes that are occurring with time in these simulations. Here, we compare the vertically integrated aerosol mass column abundance for each of the coagulation test simulations. In the nominal mode, shown in the top panel, we see that after some initial instability through the first two years of the simulation, the cloud eventually calms down to an approximately steady state, with few perturbations (none, in fact, for the more equatorial latitudes). A similar behavior is seen in the middle panel, showing the coagulation-suppressed simulation, but note that we do not see the higher latitude profiles here because their day-to-day excursions in aerosol distribution and vapor concentration became too great for model stability. In the lower panel, we see that the polar profile exhibits variations at the magnitude of a factor of two in integrated aerosol mass column abundance, and timescales of tens to hundreds of days. These significant variations continue throughout the four simulated years and show no signs of diminishing (though they also show no signs of increasing in magnitude, either). Furthermore, we see much smaller (in both amplitude and period) day-to-day variations in the ‘mid2’ and ‘pol1’ profiles, but we also see a much larger aerosol mass column abundance achieved as a quasi-steady state. In fact, the values obtained by these two simulations are comparable to the LCPS results if the Mode 3 population is assumed to be exactly as reported by Knollenberg and Hunten (1980), without the Pollack et al. (1980) reduction by a factor of three. However, the particle size distributions (Fig. 22) are not consistent with such a scenario. It is important to note that we do not claim that this variation is an expected behavior of the Venus clouds. In fact, the incompatibility between the characteristics of the modeled variability just described and the observed variability precludes this. However, that this instability arises and remains throughout the simulation indicates that the change in this physical parameter, in conjunction with the other processes at work, does appear to provide a somewhat destabilizing effect on the distribution of the aerosol.

Upon closer inspection, we can investigate the trends of the ‘pol2’ mass loading and effective radius during the course of some of these excursions. Also in Fig. 21, we show the mass loading and the effective radius of the aerosols near cloud base in the ‘pol2’ profile, for 50 days on either side of simulation day number 1000. Here, we see that the changes in effective radius are very rapid, occurring essentially within a single day as the population of small, newly activated droplets skyrockets. The mass loading climbs soon afterward as these droplets grow via condensation. The particles, initially too small to fall out of the cloud on a rapid enough timescale compared with the growth processes, gradually grow in size, and the effective radius begins to recover. However, this growth results in some of the aerosol mass being removed from the cloud (at least at this altitude), and a reduction in the mass loading is seen. In this case, there is another burst of aerosol activation before the cloud can completely recover its initial state, and the process begins anew.

In Fig. 22, we show contour plots of the particle size distributions for this simulation. On the one hand, we see many similarities between this and the nominal case. For example, the clear delineation between the CCN population centered around $0.1\,\upmu \hbox {m}$, which occupies altitudes from 40 to 60 km; the photochemical upper cloud production region, exhibiting particles a bit larger than $1\upmu \hbox {m}$, occupying altitudes from about 60 km upward, diminishing in number with altitude; and the larger particles in the condensational cloud, growing from sizes around $1\upmu \hbox {m}$  around 60 km to sizes approaching $10\upmu \hbox {m}$ at cloud base, which varies with latitude. However, the ‘mid2’ and ‘pol1’ profiles are distinctly different than their nominal case counterparts. In each, the number of particles in the upper clouds is enormously larger, exceeding the scale chosen for these plots throughout the paper. The sizes of these particles are also a bit smaller than the nominal case, peaking around $0.3\upmu \hbox {m}$. Second, the number of particles present in the condensational cloud between about 45 and 55 km is much larger than the nominal case. The location of the peak of this distribution is a bit smaller in radius than before, at about 2–3 $\upmu \hbox {m}$, but the number of the largest particles in the distribution (*i.e.*, the extent of the breadth of the distribution in radius space) is comparable to the previous simulation. Finally, the condensational cloud region is almost completely devoid of involatile particles that can serve as cloud condensation nuclei (CCN). The CCN appear to have been almost entirely scavenged by the sulfuric acid droplets. This likely has contributed to the redistribution of volatile mass, as the larger number of potential activation sites results in a more even distribution of mass, hence smaller and more numerous droplets. Another consequence of this is that the larger number of cloud droplets overall results in a larger number of droplets able to reach higher altitude via eddy diffusion in our model, possibly providing a source for observed enhancements of sulfuric acid vapor at high altitudes (Zhang et al. 2012). As to the question of why this should occur only at mid-latitudes, the answer at this point would be mere speculation. But, one possibility is that it is the result of the omission of the large-scale dynamics such as the meridional circulation. At these latitudes (between about 45$^\circ$ and 75$^\circ$), the meridional circulation is expected to reach its poleward terminus and begin to drive a downward flow. This would have significant effects on our simulated cloud. Another possibility is that the simulated cloud base is located below the fixed region of increased eddy diffusion coefficient that identifies the convective region. Hence, any volatiles deposited to these altitudes to evaporate require longer timescales to be returned by eddy diffusion to the condensation region. This is another aspect of the model that will be improved when we restore the radiative heating and cooling to determine the temperature profile and convection in the simulations.

**Fig. 23:**
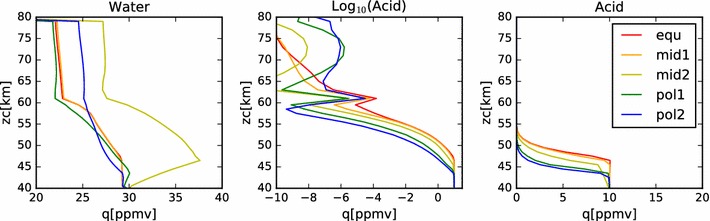
Mixing ratios of water, acid and log(acid) for coV

In Fig. 23, we show the mixing ratios for the variable coagulation simulation. We do not show the coagulation-suppressed simulation because the equatorial profiles almost exactly match those seen in the nominal simulation. The behavior of the mixing ratios is similar to the previous simulations with one clear exception. Namely, the water vapor mixing ratio in the ‘mid1’ profile has increased significantly within the condensational cloud.

This accumulation of water is likely the result of an evaporation of the cloud droplets that are falling into drier regions of the atmosphere. In fact, it is likely that, because this happens so early in the simulation, it also is simply an effect of a ‘ringing’ of the model as it moves from the initial state to a steady state. However, in making such a large excursion, this particular simulation seems to have found a new, slightly different steady state than what is usually obtained. Note that if these simulations included the full set of sulfur chemistry reactions at all altitudes, then this water vapor would very rapidly be converted into $\hbox {H}_2\hbox {SO}_4$ by reaction with the abundant $\hbox {SO}_x$, which exhibits a strong vertical gradient, increasing with depth at these altitudes. However, since we limit the acid-forming reaction to a single altitude level (60–62 km), the water is left to accumulate and stagnate here. Future simulations that explore rapid timescale behavior in the Venus clouds will be needed to elucidate this phenomenon.

## Conclusions

This paper presents the results of a series of microphysics simulations of the aerosols in the Venus atmosphere, between the altitudes of 40 and 80 km. The final model incorporates all of the relevant cloud microphysical processes (condensational growth and evaporation, coagulation, sedimentation, and eddy diffusion transport), in addition to a simplified photochemistry parameterization and a binary homogeneous nucleation parameterization. In general, the simulated cloud in all cases is in good agreement with existing in situ and remote measurements of the Venus clouds.

In the simulations looking only at the Condensational Cloud below 60 km, we find that a complete removal of the upper cloud boundary condition has only a minimal effect on the Condensational Cloud mass and particle size distributions. Hence, the Condensational Cloud is reasonably decoupled from the Photochemical Cloud on short timescales relative to those that govern the subcloud reservoir of the sulfuric acid.

In the chemistry sensitivity tests, we found that there is a fairly small effect on the mass and particle sizes of the Condensational Cloud at low latitude; and a comparably larger effect at higher latitudes. However, this is possibly a result of the simplifying assumptions made in the photochemical production parameterization of the earlier chemistry-free simulations that are more consistent with the low latitude simulation than the high latitudes. That is, this difference likely tells us more about the inaccuracy of the high latitude 40–60 km simulations than the accuracy or inaccuracy of those simulations including chemistry. Future work will include a more realistic and interactive treatment of the relevant chemistry.

In all cases, the total mass of the Photochemical Cloud is deficient relative to the observations. The current suite of simulations is insufficient to discriminate whether this is the result of errors in the chemistry assumptions, errors in the particle microphysics assumptions, or due to the neglect of the radiative-dynamical feedback in this suite of simulations.

Temperature-dependent coagulation has been shown to have the potential to lead to a drastically different cloud distribution. While the range of mass loading, column abundance, and effective radius are all consistent with accepted bounds of previous observations, the specifics of the distributions do not appear to match previously retrieved estimates of variability. However, sensitivity to these parameters is demonstrated, and perhaps future improvements to the relevant microphysics will improve the predictions.

The nature and distribution of the potential CCN is shown to be of significant importance to determining the cloud particle distribution. The effects of nucleation and activation are tremendous. Future study and characterization of the CCN are necessary to understand the clouds of Venus, perhaps from an in situ platform.

Changes in the applied chemistry parameters sometimes led to model instability. This was usually driven by new particle formation and could be related to limitations of the assumed binary homogeneous nucleation parameterization used here. The author is aware of an ongoing effort to improve the binary homogeneous nucleation parameterization to wholly enclose the parameter space of the Venus atmosphere that contains aerosols (Määttänen, personal communication). However, since the likelihood and process of heterogeneous nucleation depend strongly on the presence and composition of involatile particles in the cloud domain, these difficulties show that an improved characterization and understanding of the CCN are necessary for a complete understanding of the Venus cloud system.

These simulations demonstrate that subtle changes in the properties of the aerosols that make up the Venus cloud system can have significant and measurable effects on the spatial distribution of the clouds. Many of the properties explored here are strong functions of particle composition. Since the composition of much of the constituents of the Venus clouds remain unconstrained, it is essential that in situ measurements be carried out in order to conclusively determine the composition of the aerosols in the Venus clouds.

The existence of an enhanced number of particles having a large range of sizes in the temperature-dependent coagulation simulation is suggestive that conditions suitable for significant charge exchange among particles that is necessary for the onset of lightning are plausible.

The observed changes can be very rapid: sometimes less than one day, but certainly exhibiting changes on scales of days to weeks. Future work will compare simulated radiances produced by these clouds to Akatsuki observations. This will enable further characterization of the structure and evolution of the Venus clouds. For example, flying an aerosol size and shape spectrometer to determine with improved spatial and size resolution the radii and habits of the Venusian aerosols will help resolve the ambiguity in the current state of understanding of the Venus cloud aerosol composition. Furthermore, such an instrument could prove insightful to the question of whether bacterial life might exist in the atmosphere of Venus, as suggested by Schulze-Makuch et al. (2004) and Limaye et al. (2017) .
